# Angiotensin-Converting Enzyme 2 (ACE2) Is a Key Modulator of the Renin Angiotensin System in Health and Disease

**DOI:** 10.1155/2012/256294

**Published:** 2012-03-20

**Authors:** Chris Tikellis, M. C. Thomas

**Affiliations:** Division of Diabetic Complications, Baker IDI Heart and Diabetes Institute, P.O. Box 6492 Melbourne, VIC 8008, Australia

## Abstract

Angiotensin-converting enzyme 2 (ACE2) shares some homology with angiotensin-converting enzyme (ACE) but is not inhibited by ACE inhibitors. The main role of ACE2 is the degradation of Ang II resulting in the formation of angiotensin 1–7 (Ang 1–7) which opposes the actions of Ang II. Increased Ang II levels are thought to upregulate ACE2 activity, and in ACE2 deficient mice Ang II levels are approximately double that of wild-type mice, whilst Ang 1–7 levels are almost undetectable. Thus, ACE2 plays a crucial role in the RAS because it opposes the actions of Ang II. Consequently, it has a beneficial role in many diseases such as hypertension, diabetes, and cardiovascular disease where its expression is decreased. Not surprisingly, current therapeutic strategies for ACE2 involve augmenting its expression using ACE2 adenoviruses, recombinant ACE2 or compounds in these diseases thereby affording some organ protection.

## 1. Introduction

**Figure 1:**
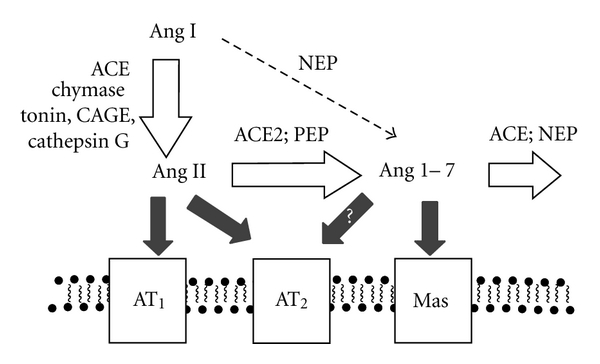
Schematic representation of the renin-angiotensin system (RAS) and the key balancing role of ACE2. Abbreviations, ACE: angiotensin-converting enzyme; ACE2: angiotensin-converting enzyme 2; NEP: neprilysin; AT1: Ang II type 1 receptor; AT2: Ang II type 2 receptor; PEP: prolyl endopeptidase; CAGE: chymostatin-sensitive angiotensin II-generating enzyme.

The renin-angiotensin system (RAS) is a signalling pathway that acts as a homeostatic regulator of vascular function [1]. Its systemic actions include the regulation of blood pressure, natriuresis, and blood volume control. However, the RAS also plays an important local role, regulating regional blood flow and controlling trophic responses to a range of stimuli. The RAS is composed of a number of different regulatory components and effector peptides that facilitate the dynamic control of vascular function, in both health and disease (Figure 1). Many of these components have opposing functions to accommodate a rapid but coordinated response to specific triggers. For example, angiotensin I (Ang I) is metabolised by the dipeptide carboxypeptidase, angiotensin-converting enzyme (ACE) to form angiotensin II (Ang II) and Ang II is metabolised by the carboxypeptidase, ACE2, producing the vasodilator, angiotensin_(1–7)_ (Ang 1–7) [2–4]. Historically, ACE and Ang II have been the key focus for clinical interventions targeting the RAS and its pathogenic actions. However, recent studies have also demonstrated the importance of ACE2 in maintaining the balance of the RAS. Indeed, in some settings, and the cardiovascular system in particular, ACE2 may be more important than ACE in regulating local levels of Ang II and Ang 1–7, and therein the balance of RAS activation. For example, we have shown that acquired or genetic deficiency of ACE2 results in increased tissue and circulating levels of Ang II [5, 6] and reduced levels of Ang 1–7 [6]. By contrast, *Ace* KO mice have modestly reduced circulating Ang II, while tissue levels are not significantly modified, possibly as substantial amounts of Ang II are generated by non-ACE pathways, while degradation pathways for Ang II are more limited [7]. This paper will specifically examine the actions of ACE2 in the body and discuss their potential role in health and various disease states.

## 2. Angiotensin-Converting Enzyme (ACE2)

ACE2 is a type 1 integral membrane glycoprotein [8] that is expressed and active in most tissues. The highest expression of ACE2 is observed in the kidney, the endothelium, the lungs, and in the heart [2, 8]. The extracellular domain of ACE2 enzyme contains a single catalytic metallopeptidase unit that shares 42% sequence identity and 61% sequence similarity with the catalytic domain of ACE [2]. However, unlike ACE, it functions as a carboxypeptidase, rather than a dipeptidase, and ACE2 activity is not antagonized by conventional ACE inhibitors [4]. The major substrate for ACE2 appears to be (Ang II) [2–4], although other peptides may also be degraded by ACE2, albeit at lower affinity. For example, ACE2 is able to cleave the C-terminal amino acid from angiotensin I, vasoactive bradykinin (1–8), des-Arg-kallidin (also known as des-Arg^10^ Lys-bradykinin) [2], Apelin-13 and Apelin-36 [9] as well as other possible targets [10]. The noncatalytic C-terminal domain of ACE2 shows 48% sequence identity with collectrin [11], a protein recently shown to have an important role in neutral amino acid reabsorption from the intestine and the kidney [12]. This is highly consistent with ACE2's actions as a carboxypeptidase, as the removed amino acid then becomes available for reabsorption. The cytoplasmic tail of ACE2 also contains calmodulin-binding sites [13] which may influence shedding of its catalytic ectodomain. In addition, ACE2 has also been associated with integrin function, independent of its angiotensinase activity.

## 3. ACE2 and Atherosclerosis

Abnormal activation of the RAS contributes to the development and progression of atherosclerotic vascular disease [14–16]. Independent and additional to the induction of systemic hypertension and vasoconstriction, Ang II has a number of direct proatherosclerotic effects on the vascular wall [17–19], including promoting inflammation [20], endothelial dysfunction [21], oxidative stress, endothelial cell, and vascular smooth muscle cell migration, growth, proliferation [22], and thrombosis. By contrast, the major product of ACE2, Ang 1–7, has a range of anti-inflammatory and antioxidant effects [23, 24] that oppose those of Ang II in the vasculature. Indeed, an infusion of Ang 1–7 is able to attenuate vascular dysfunction and atherosclerosis in genetically susceptible *apolipoprotein E* knockout (*apoE* KO) mice [25], possibly by increased activation of the Mas receptor and the type 2 angiotensin receptor (AT_2_). It is thought that the balance of Ang II and Ang 1–7 represents an important driving factor for vascular disease progression. Consequently, ACE2 is also likely to play an important role in atherosclerotic plaque development. Certainly, ACE2 expression is reduced in established atherosclerotic plaques [26] and in proatherosclerotic states, such as diabetes [27]. However, direct evidence for ACE2 in the development and progression of atherosclerotic plaques has only recently become available [5].

**Figure 2:**
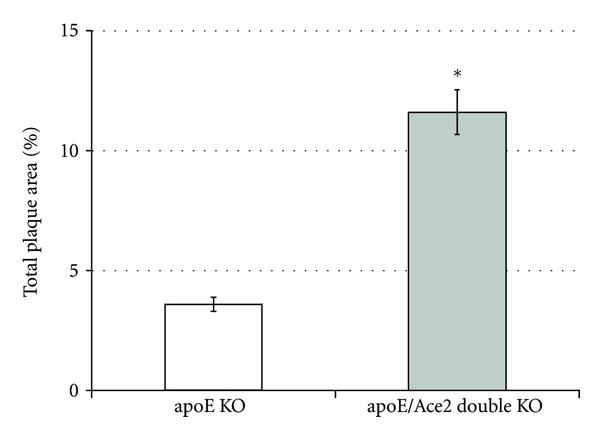
Increased plaque area accumulation in the aorta of *Apoe/Ace2* double KO mice when compared to control *Apoe* KO mice [5]. *vs control *Apoe* KO mice *P* < 0.05.

We have shown that in *apoE* KO mice, deficiency of ACE2 is associated with increased plaque accumulation (Figure 2), comparable to that observed following angiotensin II infusion [19]. This possibly relates to an increased proinflammatory responsiveness [5], as leukocyte recruitment and adhesion to the nascent atherosclerotic lesion is generally regarded as one of the first steps toward plaque formation. While a healthy endothelium does not in general support binding of white blood cells, we show that the aortic endothelium of *apoE/Ace2* double KO mice shows increased adhesion of labeled leukocytes [5]. In addition, genetic ACE2 deficiency is associated with upregulation of putative mediators of atherogenesis, such as cytokines and adhesion molecules. The role of the RAS in these actions is further emphasized by the finding that RAS blockade is able to prevent atherogenesis in *apoE/Ace2* double KO mice. Such data emphasize the potential utility of ACE2 repletion as a strategy to reduce atherosclerosis, particularly in combination with ACE inhibition and other interventions to reduce activation of the RAS (see below).

## 4. ACE2 and Hypertension

Activation of the RAS is known to be a key mediator of hypertension, and interventions to block RAS activation are the most widely used of all blood pressure lowering agents. The antihypertensive efficacy of these agents is partly mediated by their ability to reduce Ang II or its signalling. However, the antihypertensive effects of conventional RAS blockade are also partly determined by the ability of both ACE inhibitors and angiotensin receptor blockers (ARBs) to increase circulating levels of Ang 1–7 [28]. Moreover, inhibiting the vascular actions of Ang 1–7 in spontaneously hypertensive rats (SHRs) receiving RAS blockade, attenuates the antihypertensive response to these agents [28, 29]. Given that the major source of Ang 1–7 is ACE2, this data suggests that ACE2, consequently influences not only the development of hypertension, but also potentially the response to its treatment. Certainly, ACE2 expression is abnormal in SHRs, in which one genetic component of this phenotype tracks to the *Ace2* locus. In addition, ACE2 deficiency is associated with modest systolic hypertension [30], although the mouse genetic background significantly alters the cardiovascular phenotype [30–33]. *Ace2* KO mice also have a heightened hypertensive response to Ang II infusion associated with exaggerated accumulation of Ang II in the kidney [30].

The RAS and ACE2 are also implicated in the pathogenesis of central hypertension. In particular, the rostral ventrolateral medulla (RVLM) is a relay point that provides supraspinal excitatory input to sympathetic preganglionic neurons in the regulation of blood pressure. In the SHRs, ACE2 expression is reduced in the RVLM [34], and persistent overexpression of ACE2 in the RVLM results in a significant attenuation of high blood pressure in this model [35, 36]. In addition, injections of the ACE2 inhibitor MLN4760 into the nucleus tractus solitarii reduce reflex bradycardia in response to the baroreceptor stimulation in rats [37], suggesting an additional role for central ACE2 in controlling baroreceptor responsiveness.

## 5. ACE2 in Heart Failure

In addition to effects on blood pressure, natriuresis and atherogenesis, the RAS plays a critical pathophysiological role in the maintaining and subsequently subverting cardiac function in the setting of progressive heart failure [38]. The cardiac RAS is upregulated in almost all models of cardiac injury, including volume overload [39], myocardial infarction [40], and heart failure [41]. As in the kidney, RAS upregulation appears to be a homeostatic response to restore cardiac function. For example, Ang II is an inotropic and growth factor for cardiac myocytes, stimulating compensatory hypertrophy [42]. Ang II is also important in left ventricular remodeling following myocardial infarction or with after-load-induced cardiac hypertrophy [43]. However, in the long term such actions lead to progressive functional loss and cardiac fibrosis [42], as the synthesis of extracellular matrix is increased by Ang II [44]. The key role of RAS activation in the development and progression of cardiac failure is supported by findings in a number of different models in which blockade of the RAS was able to attenuate or prevent cardiac damage, independent of blood pressure lowering [45].

**Figure 3:**
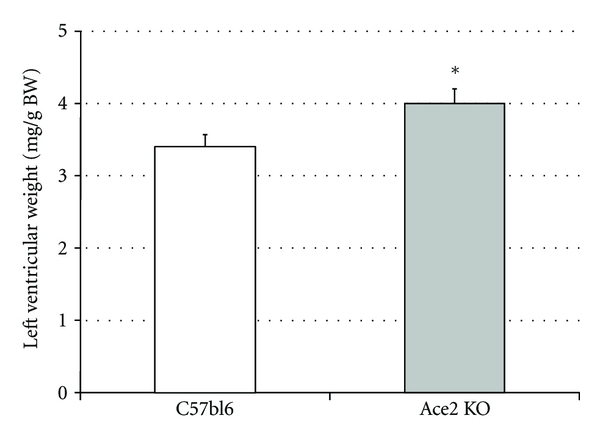
Increased LV mass in *Ace2* KO mice versus C57bl6 mice (unpublished data). *vs control C57Bl6 mice, *P* < 0.05.

In the heart, ACE2 represents the primary pathway for the metabolism of Ang II [46, 47]. ACE2 deficiency in mice results in early cardiac hypertrophy (Figure 3) [32] and accelerates adverse postmyocardial infarction ventricular remodeling [48]. Furthermore, this appears to be through the activation of the NAPDH oxidase system with the p47(phox) subunit playing a critical role [49]. In some, but not all models, ACE2 deficiency also results in progressive cardiac fibrosis with aging and/or cardiac pressure overload [33, 50, 51]. Again, these changes are reversed following treatment with ACE inhibitors or AT_1_ receptor blockers [33, 50, 51] suggesting that the balance of ACE and ACE2 in the heart is an important driving factor for progressive cardiac disease.

## 6. ACE2 and Chronic Kidney Disease (CKD)

**Figure 4:**
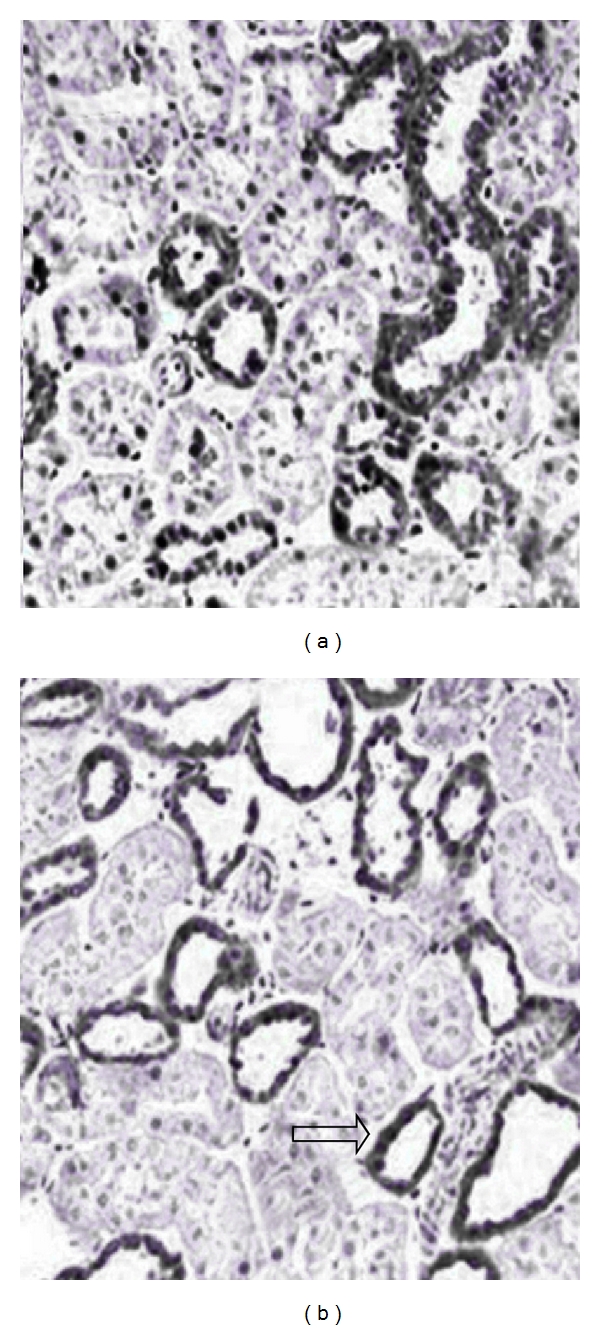
Reduced ACE2 expression (arrows) in renal cortical tubules of diabetic mice (b) when compared to control mice (a) [27].

The RAS also plays an important role in renal physiology and pathophysiology. In the adult kidney [2], ACE2 is predominantly expressed in the proximal tubule at the luminal brush border. Despite the presence of unopposed ACE activity and elevated Ang II levels, both kidney function and renal development are normal in the *Ace2 *knockout mouse [33]. By comparison, *ACE*,* angiotensinogen*, and* AT1* receptor deficiency results in a number of alterations in kidney morphology [52]. This suggests that, at least in the healthy state, ACE2 may have a limited role in regulating renal development. However, the actions of ACE2 appear to come into its own in states of RAS activation. This is much like Ang 1–7, its major product, which shows very limited renal effects in the healthy state but profound benefits in the diabetic kidney and other states associated with renal damage and activation [10, 53]. For example, ACE2 deficient mice have been reported to show increased age-related glomerulosclerosis in susceptible mouse models [54] and enhanced renal Ang II-induced renal oxidative stress, resulting in greater renal injury [55]. Similarly, in the diabetic kidney, downregulation of tubular ACE2 (Figure 4) [27] is associated with albuminuria or tubular injury, while further inhibition of ACE2 results in augmented renal damage [56, 57]. Indeed, in most forms of CKD, including diabetes, expression of ACE2 has been reported to be reduced in tubules. However, some studies have reported that glomerular ACE2 expression may be increased in human kidney disease [58]. It is possible that this differential expression pattern of glomerular and tubular ACE2 is an important determinant for progressive renal disease.

## 7. ACE2 and the Lung

RAS activity is intrinsically high in the lung, which is a major source of ACE and therefore a major site of systemic Ang II synthesis. ACE2 is also highly expressed in the lung. Pulmonary ACE2 appears to have a role in regulating the balance of circulating Ang II/Ang 1–7 levels. Ang II induces pulmonary vasoconstriction in response to hypoxia, which is important in preventing shunting in patients with pneumonia or lung injury [59]. Locally increased Ang II production also triggers increasing vascular permeability facilitating pulmonary edema [60]. In Acute respiratory distress syndrome (ARDS), the RAS appears crucial in maintaining oxygenation, possibly as widespread lung injury would otherwise result in complete pulmonary shutdown. Certainly in ARDS models, ACE2 knockout mice displayed more severe symptoms of this disease compared with wild-type mice [60] while overexpression appears protective (see below). Interestingly, ACE2 protein also appears to be the entry-point receptor for the severe acute respiratory syndrome (SARS) coronavirus [61, 62].

## 8. Replenishing ACE2 as a Potential Therapeutic

Given the key role of ACE2, degrading Ang II and generating Ang 1–7, a number of studies have explored its potential as a treatment strategy using human recombinant ACE2 (rhACE2) or adenoviral (Ad)-ACE2 in animal disease models. For example, overexpression of ACE2 in human endothelial cells attenuates Ang II-induced oxidative stress and subsequent increase in monocyte adhesion [63]. Similarly, in rabbits, a recombinant ACE2 expressing vector stabilized atherosclerotic plaques induced by balloon injury to the abdominal aorta [64]. Treatment with a lentiviral vector containing ACE2 resulted in lower blood pressure in hypertensive mice [65, 66] or following an Ang II infusion [67]. Strategies to upregulate or replenish ACE2 are thought to be beneficial in diabetic nephropathy. For example, in diabetes the replenishment of ACE2 with rhACE2 in a mouse model of type 1 diabetes attenuated diabetic kidney injury as well as reducing in blood pressure [68]. The use of (Ad)-ACE2 has had similar beneficial effects in streptozotocin-induced diabetes, where it was shown to attenuate glomerular mesangial cell proliferation, blood pressure, oxidative stress, and fibrosis [69].

In contrast to these studies, the potential utility of ACE2 supplementation in cardiac disease remains controversial. The expression of ACE2 in the failing human heart is generally increased [70–72], consistent with the finding of elevated levels of Ang 1–7 in the same setting [73]. More importantly, overexpression of ACE2 in cardiac myocytes resulted in conduction disturbances by 2 weeks of age, ultimately leading to lethal ventricular arrhythmias and severe fibrosis [74, 75]. This may be because ACE2 is not normally expressed in high levels in myocytes, although it is present in the endocardium and other cardiac cells. However, other studies using transgenic overexpression of cardiac ACE2 have demonstrated partial protection in the heart from ischemia-induced heart failure [76]. Indeed, more recent studies using rhACE2 have shown beneficial cardiac effects [77]. However, the indication for ACE2 that appears most likely to be first tested in the clinic is the treatment of ARDS. In murine models, treatment with catalytically active recombinant ACE2 protein improved the symptoms of acute lung injury in wild-type mice as well as in ACE2 knockout mice [60]. Clinical trials in this often fatal condition are now underway.

Perhaps, the most clinically interesting, however, is the potential for rACE2 to augment the vasculoprotective effects of ACE inhibition or ARBs, in the millions of patients that take these agents, worldwide. In theory, this would be achieved by preventing feedback escape for RAS blockade or enhancing the generation of Ang 1–7, and subsequent signaling through the Mas receptor and or AT_2_ receptor. Certainly, ACE2 inhibition attenuates the effects of RAS blockade, both in vitro [78] and in vivo [6]. But could rACE2 make the response to conventional RAS blockade more effective or durable? The problem is that conventional RAS blockade is highly effective in animal models of vascular and renal disease, meaning that it is difficult to explore the potential for further improvements. However, chronic intravenous infusion of ANG-(1–7), or the nonpeptide mas receptor agonist, AVE-0991, are able to improve salt-induced suppression of endothelium-dependent vasodilatation in the mesenteric arteries of male Sprague-Dawley rats, and these actions are not modified by the angiotensin receptor blocker, losartan [79], suggesting that the effects of enhancing the Ang 1–7 mas axis may be beneficial, even in the setting of conventional RAS blockade. Although it enhances the generation of Ang 1–7, whether rACE2 can also provide synergistic benefits, remains to be established.

## 9. ACE2 Augmenters: A New Kind of Intervention

Rather than providing exogenous ACE2, an alternative approach for augmenting ACE2 has been to increase its endogenous expression. For example, in hypertensive SHRs, all-trans retinoic acid, which increases ACE2 expression, lowers blood pressure levels, and prevents vascular damage [80]. Unfortunately retinoic acid has broader actions that make its potential utility as a therapeutic limited. However, compounds that increase activity of ACE2 could also be beneficial as a treatment in conditions where ACE2 activity is decreased. One exemplar is xanthenone (XNT). This molecule was selected following structure-based screening on compounds that would stabilize the activated form of ACE2, thereby enhancing its catalytic efficacy [81]. In experimental studies, this compound has been shown to enhance ACE2 activity in a dose-dependent manner and significantly decreased blood pressure in both SHRs rats and wild-type WKY rats [81]. Furthermore, improvements in cardiac function and reversal of myocardial, perivascular, and renal fibrosis in the SHRs were also observed [81, 82]. XNT has also shown promise in treating pulmonary hypertension (PH). For example, in a rat model of PH, treatment with XNT was shown to reduce elevated right ventricular systolic pressure, right ventricular hypertrophy, increased pulmonary vessel wall thickness, and interstitial fibrosis [83]. In a model of thrombus formation using SHRs and WKY rats, XNT has also shown antithrombotic action, reducing platelet attachment, and reducing thrombus formation [84]. This compound will not come to clinical trials because of issues of solubility that restrict its formulation. However, other drugs of the same class may prove more suitable.

## 10. Conclusion

ACE2 is an integral component of the RAS. It is highly expressed in the vasculature, the kidney, lungs, and heart where its actions on peptide signals balance and offset those of ACE. Its actions appear critical in a variety of disease states, including hypertension, diabetes, ageing, renal impairment, and cardiovascular disease. ACE2 deficiency leads to modest physiological changes. However, in states of RAS activation, the loss of ACE2 appears far more important in the development and progression of disease. By contrast, augmentation of ACE2 expression, either directly with recombinant ACE2 or indirectly via agonists like XNT, may have important benefits relevant in the treatment of a range of conditions.
